# The implications of signaling lipids in cancer metastasis

**DOI:** 10.1038/s12276-018-0150-x

**Published:** 2018-09-21

**Authors:** Xiangjian Luo, Xu Zhao, Can Cheng, Namei Li, Ying Liu, Ya Cao

**Affiliations:** 1 Key Laboratory of Carcinogenesis and Invasion, Chinese Ministry of Education, Xiangya Hospital, Central South University, Changsha, Hunan 410078 PR China; 2 Cancer Research Institute, School of Basic Medicine, Central South University, Changsha, Hunan 410078 PR China; 3 Key Laboratory of Carcinogenesis, Chinese Ministry of Health, Changsha, Hunan 410078 China; 4 Department of Medicine, Hunan Traditional Chinese Medical College, Zhuzhou, Hunan 412000 China; 5 Research Center for Technologies of Nucleic Acid-Based Diagnostics and Therapeutics in Hunan Province, Changsha, Hunan 410078 PR China

## Abstract

Metastasis is the most malignant stage of cancer. Lipid metabolic abnormalities are now increasingly recognized as characteristics of cancer cells. The accumulation of certain lipid species, such as signaling lipids, due to the avidity of lipid metabolism may be a causal factor of tumor malignant progression and metastatic behavior. In this review, we first describe signaling lipids implicated in cancer migration, invasion and metastasis. Next, we summarize the regulatory signaling hubs of lipid anabolic and catabolic metabolism. We then address lipid-rich circulating tumor cells (CTCs) and the lipid composition of exosomes budded off from tumor cells. We also present advances in targeting the regulatory hubs of lipid metabolism and signaling lipids in cancer therapy. Given the complexity of metabolic disorders in cancer, the development of significant portfolios of approaches to target signaling lipids by the integration of multiple chemical modulations, as well as molecular imaging modalities, should offer promising strategies for cancer therapy.

## Cancer treatment: Targeting or Modifying lipids with signaling role

Lipid molecules with cellular signaling roles are increasingly recognized as playing a role in cancer metastasis, the dangerous spread of cancer cells beyond a primary tumor, and these lipids may offer new targets for anti-cancer treatments. Researchers in China led by Xiangjian Luo and Ya Cao at the Central South University in Changsha review the involvement of signaling lipids in cancer. The signaling lipids are a structurally diverse range of molecules that can regulate metabolic processes which become disrupted in cancer. They can act within cells and also when released from some cells to interact with others. Some of the lipids are released in tiny membrane-bound sacs called exosomes. Modifying the lipid content of exosomes, or purifying and then re-administering exosomes to deliver drugs, could offer promising options for developing novel treatments for cancer.

## Introduction

Metabolic reprogramming is now acknowledged as a core hallmark of cancer, characterized by functional dependence on glucose and glutamine catabolic pathways. Tumors often share a common feature of uncontrolled cell proliferation; therefore, they must efficiently produce biomass components and energy for expansion and further dissemination^1–4^.

Lipid metabolic abnormalities are now increasingly recognized as a signature of cancer cells^5–7^. Highly proliferative cancer cells show enhanced lipid avidity by either increasing the uptake of exogenous lipids and lipoproteins or upregulating de novo lipid synthesis^5^. Activation of a variety of oncogenic pathways deregulates lipid metabolic processes, leading to the accumulation of certain lipid species such as signaling lipids. These bioactive lipids can serve as secondary signaling messengers to coordinate signal transduction cascades and to modulate a variety of carcinogenic processes, including cell proliferation, survival, chemoresistance and metastatic formation. Moreover, in the tumor microenvironment, noncancerous cells, such as endothelial cells, inflammatory cells, immune cells, fibroblasts and adipocytes, also play crucial roles in tumor expansion and malignant progression. Lipid autacoids, which are mainly composed of signaling lipids, can potentially target different cellular components in tumor microenvironments and conduct intercellular communication between cancerous and noncancerous cells^8^.

Tumor metastasis remains the major cause of cancer-related mortality, highlighting the importance of exploring new strategies to prevent and control tumor metastasis^9^. Current research on metabolism has been mostly focused on the primary tumor, while metabolic adjustments during each step of metastasis have received less attention^10^. In this review, we first discuss signaling lipids implicated in cancer migration, invasion and metastasis. Next, we summarize the regulatory hubs of anabolic and catabolic lipid metabolism. We address lipid-rich CTCs and the lipid composition of exosomes budded off from tumor cells. We also present advances in targeting the regulatory hubs of lipid metabolism and signaling lipids in cancer therapy (Table 1)^11–27^.

**Table 1:** Pharmacological tools to manipulate oncogenic regulatory pathways and lipid mediators associated with cancer metastasis

	Category	Target	Compound	Mechanism	References
		ACC	**Soraphen A**	ACC inhibitor	Beckers et al. 2007 [11]
		AMPK	Metformin	Activates AMPK, FDA approved	Pollak 2012 [12]
	Regulatory pathways of signaling lipid metabolism	PI3K	GDC-0326	p110α PI3K inhibitor	Soler A et al. 2016 [13]
				PI3K inhibitor	
		PI3K/mTOR	NVP-BEZ235		Xie G et al. 2017 [14]
				mTOR inhibitor	
		SREBP	Fatostatin	SCAP inhibitor	Kamisuki S et al. 2009 [15]
Signaling lipids	FABPs	FABP4	Carbazole butanoic acid	FABP4 inhibitor	Wang YT et al. 2016 [16]
			Aryl sulfonamide	FABP4 inhibitor	Wang YT et al. 2016 [16]
		FABP5	Pyrazole	FABP5 inhibitor	Wang YT et al. 2016 [16]
	Leukotrienes	Alox5	zileuton	Alox5 inhibitor, LT↓	Wculek SK et al. 2015 [17]
		LOX	NDGA	LOX ↓	Koontongkaew et al. 2010 [18]
	Prostaglandin	COX-2	Indomethacin	COX-2↓	Galfi et al. 2005 [19]
			Celecoxib	COX-2↓	Wang D et al. 2015 [20]
		PGD2	15-dPGJ 2	Akt ↓, PPARγ ↑	Shin et al. 2009 [21]
			PGJ2 /15-dPGJ2	PPARγ ↑	Chinery et al. 1999 [22]
		PGE2	ONO-AE3-208	EP4 ↓	Yang et al. 2006 [23]
			Curcumin	PGE 2 ↓	Lev-Ari et al. 2005 [24]
			Fish oil	PGE 2 ↓	Mund et al. 2007 [25]
			EPA	PGE 2 ↓	Petrik et al. 2000 [26]
		PGI2	Olive oil	6-keto PGF 1α ↓	Petrik et al. 2000 [26]
	Sphingolipids	SPHK1	FTY720	SPHK1 inhibitor, S1P↓	Patmanathan SN et al. 2016 [27]

ACC, acetyl-CoA carboxylases; Alox5, arachidonate 5-lipoxygenase; AMPK, AMP-activated protein kinase; COX, cyclooxygenase; FABPs, fatty acid-binding proteins; LOX, lipoxygenase; mTOR, mammalian target of rapamycin; PG, prostaglandin; PI3K, phosphoinositide 3-kinase; SREBP, sterol regulatory element-binding proteins; SPHK1, sphingosine kinases

## Signaling lipids associated with cancer metastasis

**Fig. 1:**
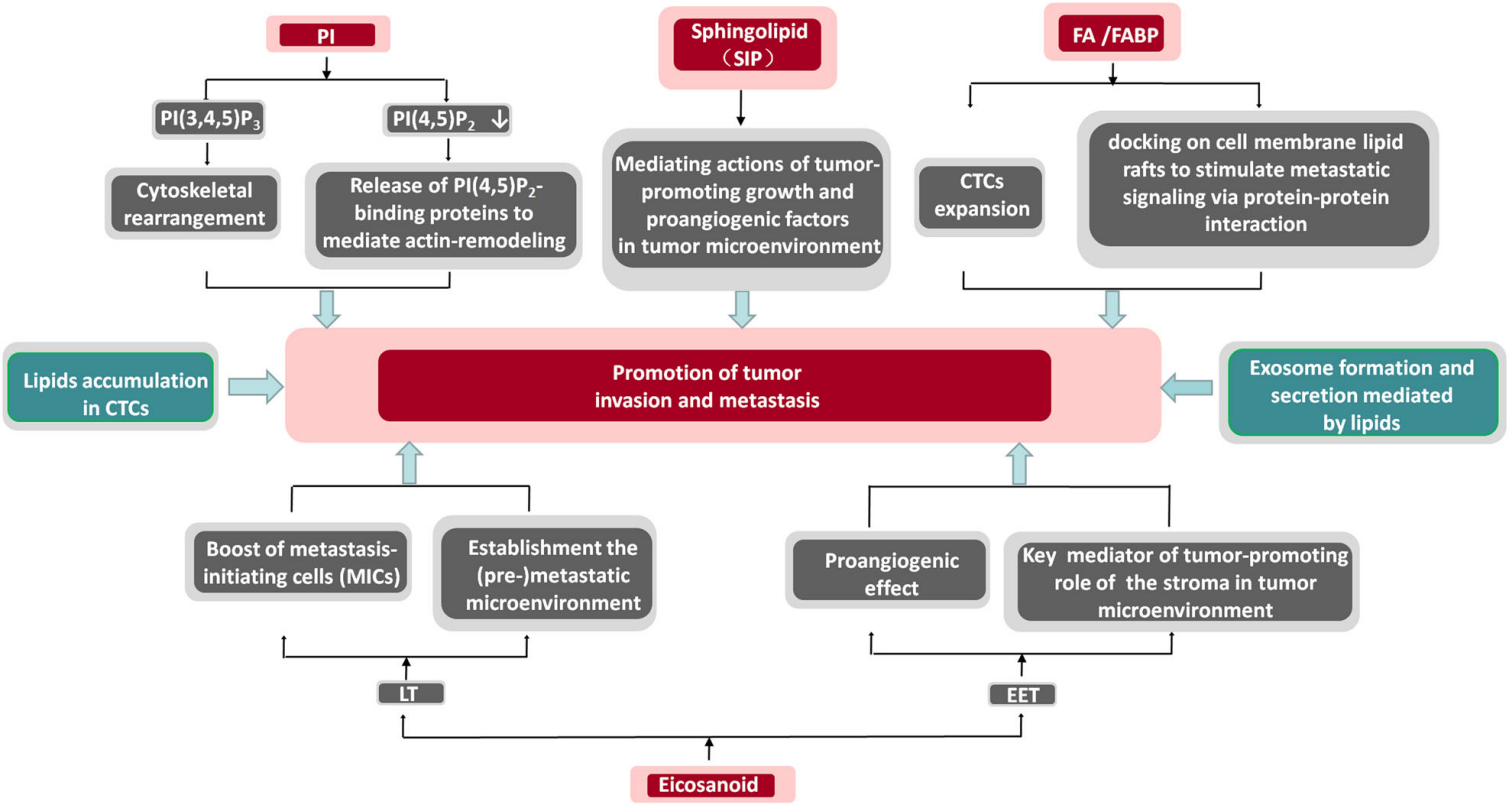
Schematic of signaling lipids implicated in cancer invasive and metastatic progression and the mechanism of action. *CTC* circulating tumor cell, *EET* epoxyeicosatrienoic acid, *FA* fatty acid, *FABP* fatty acid-binding protein, *LT* leukotriene, *PI* phosphatidyl inositides, *S1P* sphingosine-1-phosphate

In cancer cells driven by oncogenic signaling or genetic mutation of critical metabolic enzymes, the balance of metabolite homeostasis is disrupted^28^. Accumulation of signaling lipids, including eicosanoids, phosphoinositides, sphingolipids, and fatty acids, alters the cellular biochemical foundation and might be a causal factor of tumor malignant progression and metastasis^29–33^ (Fig. 1).

### Eicosanoids

Arachidonic acids are metabolized through the cyclooxygenase, lipoxygenase (LOX) and P450 epoxygenase (EPOX) pathways to generate eicosanoids, which include prostanoids, leukotrienes, hydroxyeicosatetraenoic acids, epoxyeicosatrienoic acids (EETs) and hydroperoxyeicosatetraenoic acids^34,35^. Since prostanoids, such as the proinflammatory PGE2, have long been documented for their prominent role in promoting tumor growth and metastasis^20,35–42^, we will focus on the leukotrienes and EET species of eicosanoids in this review.

### Leukotrienes (LTs)

LTs are generated by Alox5 (arachidonate 5-lipoxygenase) and are primarily produced in stimulated leukocytes. Although in comparison to prostanoids, much less is known about the involvement of proinflammatory LTs in tumor progression, emerging evidence suggests that LTs might have an important role in the establishment of premetastatic microenvironments.

Wculek et al. reported that neutrophil-derived LTs selectively expand the subpool of breast cancer cells with high tumorigenic potential and aid the colonization in a secondary organ site^17^. LTs, mainly leukotriene B4 and the cysteinyl leukotrienes C4, D4 and E4 (LTC/D/E4), boosted the heterogeneity of cancer cells, favoring metastasis-initiating cells (MICs, the CD24^+^CD90^+^ population), and led to increased metastatic competence of total breast cancer cells. Moreover, cells expressing LT receptors were shown to be enriched among MICs. Pharmacological inhibition of LOX5 by zileuton blocked LT production and impaired neutrophil prometastatic activity; consequently, there was reduced human breast cancer progression to the lungs. Additionally, selective inhibition of LOXs with NDGA (nordihydroguaiaretic acid) in colorectal cancer cells also decreased the invasive capacity of the cells via inhibition of the activities of the matrix metalloproteinases MMP-2 and MMP-9^18^. Considering that leukocytes can act as the main component and driver of metastatic establishment within the premetastatic niche in a LT/LOX5-dependent fashion, targeting the noncancer-cell component of tumor microenvironments, as well as the lipid-metabolizing enzymes and proinflammatory signaling lipids, might offer novel therapeutic approaches to limit cancer metastatic progression.

### Epoxyeicosatrienoic acids (EETs)

Arachidonic acid production is catalyzed by cytochrome P450 (CYP) epoxygenase to generate EETs, which include four regioisomeric expoxyeicosatrienoic acids, 5,6-EET, 8,9-EET, 11,12-EET and 14,15-EET. EETs are mainly secreted by endothelial cells and are metabolized by soluble epoxide hydrolase (sEH)^43,44^. Although EET receptors have not been fully identified, multiple pathways, including the GPCR/PPAR/RXR, VEGF, EGFR, tumor necrosis factor α (TNFα) and matrix metalloproteinase (MMP) pathways, are involved in the mechanism through which EETs stimulate tumor growth, angiogenesis and metastasis^45–47^.

Elevated EET levels in multiple tumor types are associated with aggressive and metastatic cell behavior. In breast cancer, upregulation of epoxygenase CYP2C8, 2C9, and 2J2 and low expression of sEH were reported to account for EET augmentation^48^. Panigrahy et al. demonstrated that in a variety of transplantable and genetically engineered mouse tumor models, endothelium-derived and systemic 14,15-EET triggered spontaneous multiorgan metastasis and escape from tumor dormancy. Downregulation of sEH resulted in increased EET levels, which subsequently stimulated the secretion of VEGF by the endothelium. Thus, the elevation of EET levels in endothelial cells at the metastatic site, and not the excessive growth of the primary tumor, led to tumor-associated angiogenesis and metastasis^49^.

Altogether, EETs can act as key mediators of protumorigenic role of the stroma in the tumor microenvironments, mainly via their proangiogenic effects. Therefore, inhibitors of EET bioactivity, such as EET antagonists, inhibitors of endothelial epoxygenases, or the overexpression of sEH may represent new intervention strategies for the metastatic progression of angiogenic cancers.

### Phosphoinositides

Abundant alteration of phosphatidyl inositides (PIs) in membranes represents a feature of cancer. PIs serve as major determinants of membrane identity and function as membrane trafficking regulators^50–53^. Among others, PI(3,4,5)P_3_ and PI(4,5)P_2_ are closely implicated in tumor cell migration and metastasis. They regulate cellular processes by recruiting, activating or inhibiting proteins at the plasma membrane to impact actin dynamics, thus causing alterations in cellular migration and metastatic capacity.

Phosphoinositide 3-kinase (PI3K) catalyzes the production of phosphatidylinositol- 3,4,5-trisphosphate (PI[3,4,5]P_3_) from its precursor PI(4,5)P_2_. Elevation of PI(3,4,5)P_3_ levels directs the guanine nucleotide exchange factors for Rho GTPases (such as Vav and Tiam) to the cell membrane, and the subsequent cytoskeletal rearrangements enhance cell migration and metastasis^54^. However, in breast cancer cells, reduced PI(4,5)P_2_ abundance in the plasma membrane enhances cellular migration and metastatic capacity. Sengelaub et al. reported that PTPRN2 and PLCβ1 enzymatically reduced plasma membrane PI(4,5)P_2_ levels, which resulted in the release of the PI(4,5)P_2_-binding protein cofilin from its inactive, membrane-sequestered state, allowing it to enter the cytoplasm. Consequently, cofilin mediated actin-remodeling and enhanced cellular migration and metastasis^51^.

Several drugs, such as NVP-BEZ235, targeting the PI3K pathway are currently undergoing phase II and III clinical trials in patients with advanced disease^13,14,55,56^. NVP-BEZ235 effectively inhibited cell migration and metastasis in vitro and in vivo, and combinations with vincristine potentiated its antimetastatic effects^56^. Since tumors with constitutively elevated PI(3,4,5)P_3_ are especially sensitive to the mTORC1 inhibitor rapamycin^55,56^, PIs such as PI(3,4,5)P_3_ may be exploited as biomarkers to identify PI3K-dependent cancers that are more likely to respond to drugs targeting PI3K/mTOR signaling.

### Sphingolipids

Sphingolipid metabolites, such as ceramide and sphingosine, act as important modulators of cell survival, angiogenesis, migration and metastasis^27,57–59^. Sphingosine-1-phosphate (S1P) is a bioactive lipid produced by the sphingosine kinases SPHK1 and SPHK2, and it can be dephosphorylated by sphingosine phosphatase or irreversibly degraded by S1P lyase (SGPL1)^60^. S1P exerts its effect via autocrine or paracrine signaling, mostly mediated by a family of five cell surface G protein-coupled receptors termed S1PR1–5^61–63^. Moreover, SP1 can bind intracellular targets such as HDAC1/2 (histone deacetylases 1/2) and NF-κB^61^.

Patmanathan et al. demonstrated that overexpression of SPHK1 and low levels of SGPL1 accounted for the augmentation of S1P levels in oral squamous cell carcinoma (OSCC). S1P protected OSCC cells from cisplatin-induced death and enhance their migration and invasion. Moreover, S1PR1 was shown to be closely associated with persistent activation of signal transducer and activator of transcription-3 (STAT3) and IL-6 expression in both tumor cells and the tumor microenvironment, both of which contributed to tumor malignant progression and distant dissemination^64^. Notably, FTY720 (2-amino-2-[2-(4-octylphenyl)]−1,3-propanediol hydrochloride), an inhibitor of SPHK1, can inhibit the proliferation and migration of a variety of cancer cell lines and suppresses tumor growth, angiogenesis and metastasis in vivo^27^.

Because S1P not only affects tumor cells but also mediates the actions of tumor-promoting growth and proangiogenic factors in the tumor microenvironment, it may be developed into a bona fide cancer target.

### Fatty acids (FAs) and lipid-binding proteins

Excessive incorporation of FAs into cancer cell membranes results in membrane phase separation, reduced cell-cell contact, and enhanced surface adhesion and tissue invasion^31–33^. Le et al. used an animal cancer model to show that mice with excess plasma FFAs due to a high fat diet (HD) exhibited early appearance of a high number of CTCs and increased lung metastasis^31^. Abundant polyunsaturated FFAs in the blood plasma induced cancer cell membrane phase separation and the polarized distribution of cellular content; these exposed cells strongly resembled CTCs isolated from HD-fed mice.

Fatty acid-binding proteins (FABPs) are a family of low molecular-mass intracellular lipid-binding proteins consisting of ten isoforms, FABP1-10. FABPs are involved in binding and storing FAs, as well as transporting them to the appropriate compartments in the cell, including the plasma membrane, nucleus, endoplasmic reticulum, mitochondria and peroxisomes^65^.

Lipids can serve as passive components of cell membranes, in which they form lipid rafts that facilitate signaling protein recruitment and protein-protein interactions to activate signal transduction pathways. Hence, FABPs, acting as chaperonins of lipids, might stimulate metastasis-associated signaling through protein-protein interactions after docking on membrane rafts. Liver fatty acid-binding protein (L-FABP) uniquely binds to ligands (e.g., long chain fatty acids) and hydrophobic molecules (e.g., cholesterol and bile acids). Ku et al. reported that L-FABP interacted with VEGFR2 on membrane rafts and subsequently activated the downstream AKT/mTOR/P70S6K/4EBP1 and Src/FAK/cdc42 pathways. This activation resulted in upregulation of VEGF-A, accompanied by an increase in both the angiogenic potential and the migration activity of hepatocellular carcinoma cells^66^. Moreover, several FABP isoforms are strongly implicated in cancer metastatic progression. High expression of FABP3 or FABP4 in non-small cell lung cancer (NSCLC) was significantly associated with advanced tumor node metastasis stage and had a negative impact on the overall survival of NSCLC patients^67^. FABP5 increased the metastasis of triple negative breast cancer in part by inhibiting EGFR proteasomal degradation and EGF-induced metastatic signaling^68^. In addition, FABP7 is involved in fatty acid metabolism and might be developed into a useful marker for the detection of metastatic melanoma^69^.

Overall, FABPs represent potential targets in cancer therapy, and FABP inhibitors could be promising cancer treatments that inhibit or reduce early-stage tumors and metastasis^16^.

## Regulation of signaling lipid metabolism

**Fig. 2:**
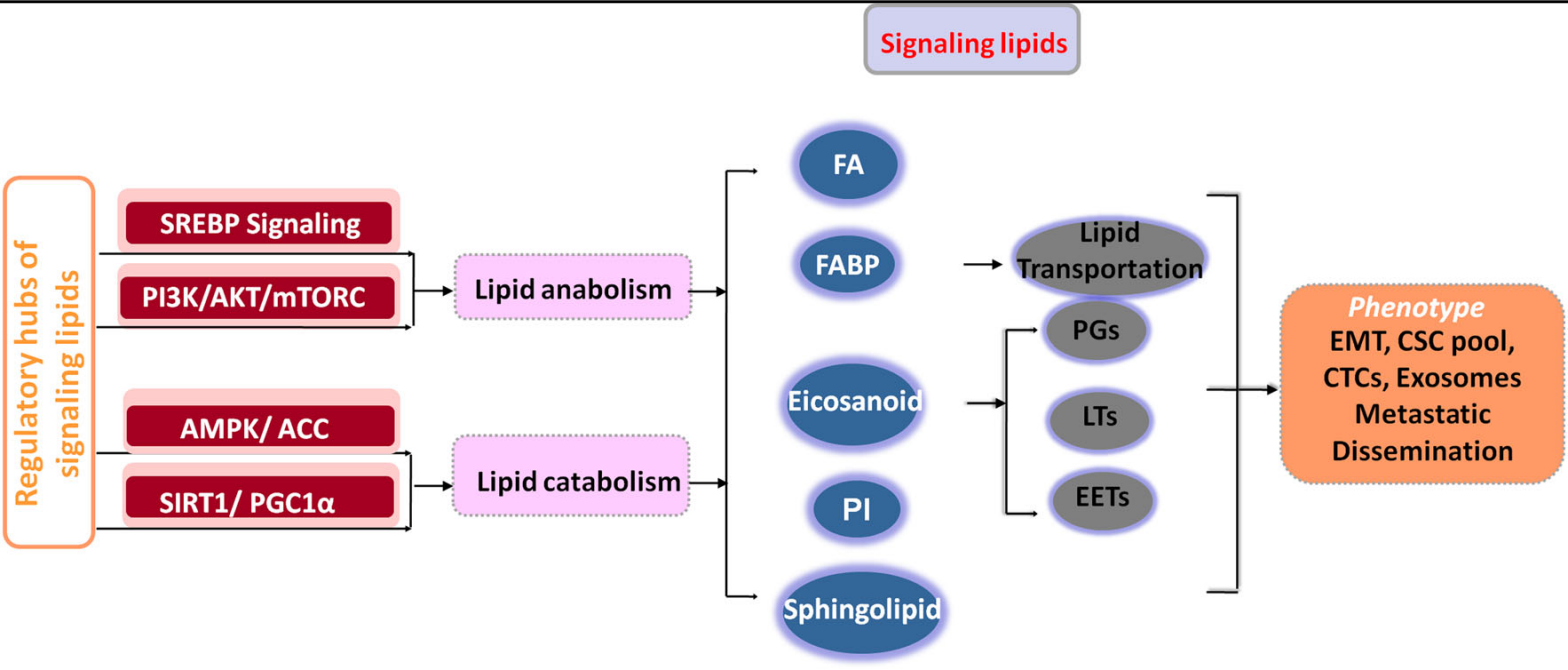
Connections among lipid regulatory pathways, signaling lipids and the invasive and metastatic phenotypes of tumor cells. SREBP signaling and the PI3K/AKT/mTORC axis are mainly responsible for lipid anabolic metabolism, and the AMPK/ACC and SIRT1/PGC1α axes contribute to lipid catabolic metabolism in tumor cells. Accumulating signaling lipids, such as FAs, eicosanoids, PIs, sphingolipids and FABPs, represent causal factors for the cancer cell EMT program, the maintenance of the CSC subpool, the increased number of CTCs and the metastatic phenotype. *CSC* cancer stem cell, *CTC* circulating tumor cell, *EETs* epoxyeicosatrienoic acids, *EMT* epithelial–mesenchymal transition, *FAs* fatty acids, *FABPs* fatty acid-binding proteins, *LTs* leukotrienes, *PGs* prostaglandins, *PIs* phosphatidyl inositides, *ACC* acetyl-CoA carboxylases, *Alox5* arachidonate 5-lipoxygenase, *AMPK* AMP-activated protein kinase, *COX* cyclooxygenase, *FABPs* fatty acid-binding proteins, *LOX* lipoxygenase, *mTOR* mammalian target of rapamycin, *PG* prostaglandin, *PI3K* phosphoinositide 3-kinase, *SREBP* sterol regulatory element-binding proteins, *SPHK1* sphingosine kinases

Metabolite homeostasis is determined by the associated metabolic pathways that synthesize or degrade metabolites. Among others, SREBP signaling, PI3K/AKT/mTORC, AMPK /ACC, SIRT1/PGC1α axes and their reciprocal dialogs serve as the regulatory hubs of signaling lipid metabolism (Fig. 2).

### SREBP signaling

Most enzymes involved in fatty acid and cholesterol biosynthesis are regulated by the sterol regulatory element-binding proteins (SREBPs), which are transcription factors in the helix-loop-helix leucine zipper family. Three SREBP isoforms, SREBP1a, SREBP1c and SREBP2, have been identified in mammalian cells. SREBP1 mainly regulates fatty acid, phospholipid and triacylglycerol synthesis, while SREBP2 controls the expression of cholesterol-synthesis genes^70^. SREBP function is modulated by protein posttranslational modifications. AMPK directly phosphorylates SREBP to prevent its proteolytic activation^71,72^. SREBP can also be phosphorylated by glycogen synthase kinase 3, resulting in protein polyubiquitination and degradation^73^.

Due to the importance of SREBP activation in signaling lipid anabolic metabolism, its overexpression is significantly associated with aggressive pathological features and has prognostic roles in multiple types of human cancer^74–78^. Genetic silencing of SREBP-2 inhibited prostate cancer (PCa) cell growth, stemness, and xenograft tumor growth and metastasis^78^. Genetic overexpression of SREBP-1 in PCa cells resulted in increased fatty acid synthase and NADPH oxidase 5 (Nox5) expression, ROS generation, fatty acid and lipid droplet accumulation. These alterations induced by SREBP-1 promoted the growth, migration, and invasion progression of PCa cells in vitro and in vivo^74^. Fatostatin was recently discovered as a specific inhibitor of SCAP (SREBP cleavage-activating protein), which is required for SREBP activation^15^. It may be developed into a novel strategy targeting lipogenesis and cholesterogenesis to treat aggressive types of cancer that have elevated lipid accumulation, undergo rapid proliferation and often develop resistance to current anticancer therapies^79^.

### PI3K/AKT/mTORC axis

The PI3K/AKT/mTORC (phosphatidylinositol 3-kinase/protein kinase B/mammalian target of rapamycin complex) axis impinges on various aspects of metabolism and impact on signaling lipid anabolic metabolism^80,81^. PI3Ks (type I) are activated by cell surface receptors and subsequently transduce the inputs into the accumulation of the signaling lipid PI(3,4,5)P_3_, which then facilitates the activation of many effectors, including the serine/threonine kinase AKT. AKT can phosphorylate ATP-citrate lyase and activate the expression of several genes involved in cholesterol and fatty acid biosynthesis^80^. SREBP is an important component of the metabolic regulatory network downstream of the PI3K/AKT/mTORC axis. Both AKT2 and mTORC1 activities have been found to be required for the induction of SREBP1c and for lipid synthesis in the liver^82,83^. In addition, the PI3K/AKT axis is implicated in the transportation and function of signaling lipids. PI3K/Akt signaling mediates the phosphorylation of the signaling lipid S1P transporter, Spns2 (spinster homolog 2), by hepatocyte growth factor and lamellipodia formation in lung endothelium cells^84^.

### AMPK /ACC axis

The AMPK/ACC axis has been proposed as a key contributor to lipid homeostasis. AMP-activated protein kinase (AMPK) is a heterotrimeric serine/threonine kinase that is activated under conditions of cellular energy shortage. Once activated, AMPK redirects lipid metabolism towards increased catabolic fatty acid oxidation and decreased anabolic lipid synthesis through the phosphorylation of acetyl-CoA carboxylases (ACCs)^85^. ACCs are responsible for the carboxylation of acetyl-CoA to form malonyl-CoA, which represents the first step in de novo lipid synthesis. In addition to ACCs, other key enzymes of lipid metabolism are known substrates of AMPK, such as Desnutrin/ATGL, whose phosphorylation facilitates the lipolytic program^86–90^. Given the important roles of the AMPK/ACC axis in the regulation of fatty acid synthesis, activation of AMPK or inhibition of ACC may develop into attractive therapeutic options in cancer types associated with fatty acid accumulation^12,85,91,92^.

### SIRT1/PGC1α axis

Sirtuins in mammals share extensive homology with the Sirt2 gene in yeast and comprise a small family with seven members, SIRT1-SIRT7. SIRT1 is the most well-characterized member of the sirtuin family and can deacetylate histone and nonhistone proteins. Through its ability to deacetylate target proteins, SIRT1 regulates lipid metabolism by interacting with certain partners such as peroxisome proliferator-activated receptor gamma coactivator-1α (PGC-1α), SREBP, PPARγ (peroxisome proliferator-activated receptor gamma), LXR (liver X receptor), Akt and AMPK in a cell context-dependent manner^35,93–96^.

The deacetylation of PGC-1α by Sirt1 has been extensively implicated in the metabolic control of signaling lipid homeostasis^93–95^. PGC-1α acts as a master transcriptional regulator of mitochondrial biogenesis, lipogenesis and fatty acid oxidation^97^. In fasting liver, the SIRT1/PGC-1α axis was activated to initiate transcription of fatty acid oxidation genes and to promote fatty acid expenditure^93^. Considering that SIRT1 and/or PGC-1α expression is highly associated with tumor invasion and metastasis^98–100^, the SIRT1/PGC-1α axis may provide an important molecular link that couples lipid metabolism to tumor malignant progression.

## Lipids implicated in CTCs

As the shedding of cells from the primary tumor into peripheral blood is a necessary step in tumor dissemination, CTCs are considered promising prognostic metastatic biomarkers^69^. Accumulating evidence supports the idea that CTCs contain a subpopulation of cancer stem cells that give rise to distant metastases^101^. Intracellular lipid content might serve as a potential biomarker of CTCs.

Coherent anti-Stokes Raman scattering (CARS) microscopy is a highly sensitive imaging technique for the visualization of lipid-rich structures. Metastatic human prostate cancer cells display rapid lipid uptake and slow lipid mobilization kinetics when incubated with human plasma spiked with palmitic acid^32^. CTCs isolated from multiple types of metastatic cancer patients exhibited strong CARS signals due to intracellular lipid accumulation^31^. Thus, in addition to the routine strategies for CTC detection, such as capturing CTCs by microposts or magnetic beads coated with specific antibodies^101^, measuring DNA shedding by CTCs^102^, and physical separation by size and density^103^, the detection of lipid-rich CTCs with label-free and nonperturbative imaging using CARS microscopy or other multimodal imaging systems may be developed as effective clinical modalities.

## Lipids in exosomes

Exosomes are small vesicular bodies (40–150 nm in diameter) released by the exocytosis of multivesicular bodies (MVBs)^104,105^. Exosomes that bud off from tumor cells might help stromal cells modulate the microenvironment and prime organs for cancer spread^106^. Lipids can mediate the formation and secretion of exosomes and contribute to their role in tumor growth and dissemination. Moreover, the composition and content of lipid species enriched in exosomes may be potential sources of noninvasive cancer biomarkers.

Several lipids and lipid-metabolizing enzymes have been shown to participate in the production and release of exosomes^107^. Among others, the levels or formation of phosphoinositides, diacylglycerol, phosphatidic acid (PA), as well as ceramide, have important roles in this process^108–110^. In prostate cancer PC-3 cells, impaired PI(3,5)P2 production by knockdown of PIKfyve or inhibition of enzyme activity increased exosome secretion and inhibited the fusion of MVB with lysosomes, which might be attributed to the ability of PI(3,5)P2 to act as an agonist of the lysosomal Ca^2+^ channel TRPML1^111^. Trajokovic et al. observed that neutral sphingomyelinase (nSMase) promotes the secretion of exosomes from Oli-neu cells triggered by ceramide formation^109^. Moreover, the regulatory mechanisms are different not only among various cell types but also among various exosome populations within a single cell line^107^. In breast cancer MCF-7 cells, the enzyme phospholipase D2 (PLD2) was required for the formation of intraluminal vesicles within a fraction of the MVBs, while inhibition of PLD2 activity attenuated only the secretion of syntenin-containing exosomes in these cells^108^.

The diverse contents of exosomes, such as nucleic acids, proteins and lipids, makes them an excellent source of noninvasive biomarkers^112–114^. Some research groups have exerted efforts to utilize lipids in urine as cancer biomarkers^115^. A study of urinary carcinoma using exosomal lipidomics revealed differences in the composition of lipid species between the carcinoma and healthy groups^115^. In addition, the effects of exosomes are not only mediated by their protein and nucleic acid cargo, but, remarkably, exosomal lipids also contribute to their bioactivity. Lombardo’s group created synthetic exosome-like lipid raft-rich nanoparticles (SELNs) that were devoid of nucleic acids and proteins^116^. In pancreatic cancer cells, SELNs activated the NF-κB/SDF-1α axis and promoted the binding of secreted SDF-1α to chemokine receptors (CXCR4) on the cell surface to further drive the Akt survival pathway^116^. Thus, exosomal lipids may enhance tumor aggressiveness, metastatic progression and drug resistance.

Altogether, lipids and lipid-metabolizing enzymes may modulate the formation and secretion of exosomes, as well as their bioactivity, and represent potential biomarkers in clinical cancer diagnosis and prognosis.

## Conclusions and perspectives

**Fig. 3:**
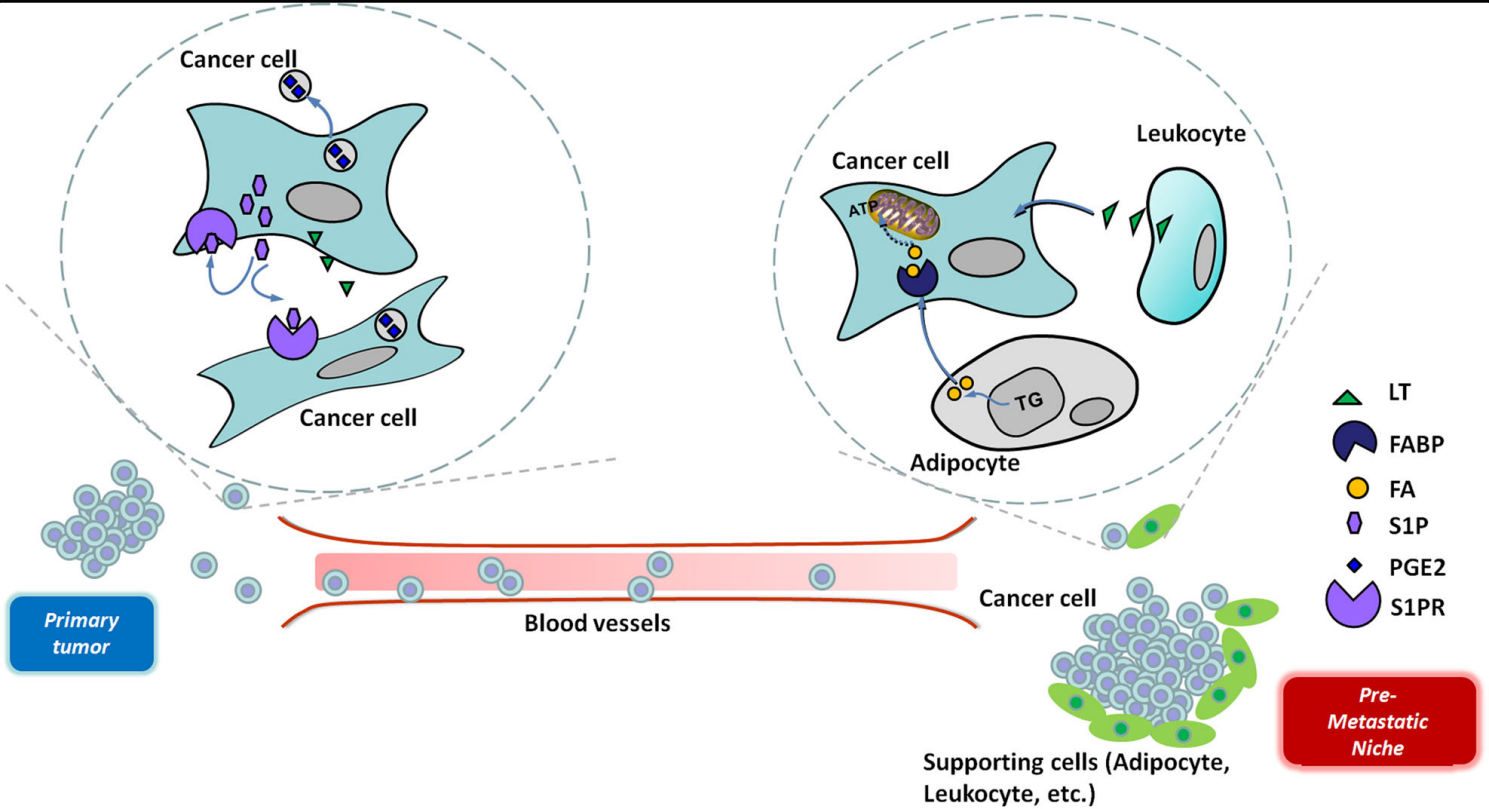
Illustrations of the signaling lipids implicated in cancer metastasis. Signaling lipids accumulate intracellularly or are secreted extracellularly to facilitate tumor cell aggressive progression and metastasis. *CTC* circulating tumor cell, *FA* fatty acid, *FABP* fatty acid-binding protein, *LT* leukotriene, *PGE2* prostaglandin E2, *S1P* sphingosine-1-phosphate, *S1PR* S1P receptor

Cancer can be characterized by the malignant and systematic dysfunction of metabolic processes. Metastasis is the most malignant stage of cancer, and lipid reprogramming may contribute to each step of metastatic formation (Fig. 3). In primary cancer cells driven by oncogenic pathways or restrained microenvironments, the lipid metabolic network is deregulated, and the balance of lipid uptake/mobilization is disrupted. Consequently, the accumulated signaling lipids may mediate intercellular communication between cancerous and noncancerous cells in the tumor microenvironment, thus facilitating the cancer cell EMT program, supporting the maintenance of the CSC subpool, and increasing the number of CTCs, leading to the acquisition of a metastatic phenotype. Moreover, lipid metabolic enzymes and signaling lipids play important roles in the regulation of exosome formation and release from cancer cells. Exosomal lipids can modulate their bioactivity in the tumor microenvironment and during distant dissemination. Furthermore, in premetastatic niches, proinflammatory signaling lipids may help establish microenvironments favorable for cancer metastasis.

Massive medicinal chemistry efforts have sought methods for the chemical modulation of lipid metabolic network nodes critical to pathological processes (Table 1). Moreover, blocking noncancerous cells in microenvironments, such as neutrophil recruitment to premetastatic sites, or decreasing proinflammatory signaling lipid levels may provide novel strategies to abrogate cancer metastatic progression. By considering exosomes as biological drug carriers that could exert specific effects on tumor environments and spreading, an understanding of how their lipid components contribute to exosomal bioactivity in recipient cells will facilitate the design of efficient exosome-based cancer therapeutics. In addition, the development of multimodal imaging CARS microscopy and CARS intravital flow cytometry may support the further elucidation of the mechanistic link between lipid-rich tumors and aggressive tumor behaviors and provide a promising modality for the label-free detection of early-stage cancer metastasis.

Given the complexity of metabolic disorders in cancer, the development of robust portfolios to target oncogenic lipids via integration of multiple chemical modulations, as well as molecular imaging modalities, should offer promising strategies for cancer therapy.

## References

[1] Hanahan D, Weinberg RA (2011). Hallmarks of cancer: the next generation. Cell.

[2] Benjamin DI, Cravatt BF, Nomura DK (2012). Global profiling strategies for mapping dysregulated metabolic pathways in cancer. Cell. Metab..

[3] Luo X. J. et al. DNMT1 mediates metabolic reprogramming induced by Epstein-Barr virus latent membrane protein 1 and reversed by grifolin in nasopharyngeal carcinoma. *Cell Death Dis*. **9**, 619 (2018). 10.1038/s41419-018-0662-2 PMC5966399 29795311

[4] Xiao L (2014). Targeting Epstein–Barr virus oncoprotein LMP1-mediated glycolysis sensitizes nasopharyngeal carcinoma to radiation therapy. Oncogene.

[5] Currie E, Schulze A, Zechner R, Walther TC, Farese RV (2013). Cellular fatty acid metabolism and cancer. Cell Metab..

[6] Luo X (2017). Emerging roles of lipid metabolism in cancer metastasis. Mol. Cancer.

[7] Tan Z (2018). Targeting CPT1A-mediated fatty acid oxidation sensitizes nasopharyngeal carcinoma to radiation therapy. Theranostics.

[8] McAllister SS, Weinberg RA (2010). Tumor-host interactions: a far-reaching relationship. J. Clin. Oncol..

[9] Cheung KJ, Ewald AJ (2016). A collective route to metastasis: Seeding by tumor cell clusters. Science.

[10] Santos CR, Schulze A (2012). Lipid metabolism in cancer. Febs. J..

[11] Beckers A (2007). Chemical inhibition of acetyl-CoA carboxylase induces growth arrest and cytotoxicity selectively in cancer cells. Cancer Res..

[12] Pollak MN (2012). Investigating metformin for cancer prevention and treatment: the end of the beginning. Cancer Discov..

[13] Soler A (2016). Therapeutic benefit of selective inhibition of p110alpha PI3-kinase in pancreatic neuroendocrine tumors. Clin. Cancer Res..

[14] Xie G (2017). Dual blocking of PI3K and mTOR signaling by NVP-BEZ235 inhibits proliferation in cervical carcinoma cells and enhances therapeutic response. Cancer Lett..

[15] Kamisuki S (2009). A small molecule that blocks fat synthesis by inhibiting the activation of SREBP. Chem. Biol..

[16] Wang YT, Liu CH, Zhu HL (2016). Fatty acid binding protein (FABP) inhibitors: a patent review (2012-2015). Expert Opin. Ther. Pat..

[17] Wculek SK, Malanchi I (2015). Neutrophils support lung colonization of metastasis-initiating breast cancer cells. Nature.

[18] Koontongkaew S, Monthanapisut P, Saensuk T (2010). Inhibition of arachidonic acid metabolism decreases tumor cell invasion and matrix metalloproteinase expression. Prostaglandins Other Lipid Mediat..

[19] Galfi P, Neogrady Z, Amberger A, Margreiter R, Csordas A (2005). Sensitization of colon cancer cell lines to butyrate-mediated proliferation inhibition by combined application of indomethacin and nordihydroguaiaretic acid. Cancer Detect. Prev..

[20] Wang D, Fu L, Sun H, Guo L, DuBois RN (2015). Prostaglandin E2 promotes colorectal cancer stem cell expansion and metastasis in mice. Gastroenterology.

[21] Shin SW (2009). 15d-PGJ2 induces apoptosis by reactive oxygen species-mediated inactivation of Akt in leukemia and colorectal cancer cells and shows in vivo antitumor activity. Clin. Cancer Res..

[22] Chinery R (1999). Prostaglandin J2 and 15-deoxy-delta12,14-prostaglandin J2 induce proliferation of cyclooxygenase-depleted colorectal cancer cells. Cancer Res..

[23] Yang L (2006). Host and direct antitumor effects and profound reduction in tumor metastasis with selective EP4 receptor antagonism. Cancer Res..

[24] Lev-Ari S (2005). Celecoxib and curcumin synergistically inhibit the growth of colorectal cancer cells. Clin. Cancer Res..

[25] Mund RC (2007). Decreased tumor growth in Walker 256 tumor-bearing rats chronically supplemented with fish oil involves COX-2 and PGE2 reduction associated with apoptosis and increased peroxidation. Prostaglandins Leukot. Essent. Fat. Acids.

[26] Petrik MB, McEntee MF, Chiu CH, Whelan J (2000). Antagonism of arachidonic acid is linked to the antitumorigenic effect of dietary eicosapentaenoic acid in Apc(Min/+) mice. J. Nutr..

[27] Patmanathan SN (2016). Aberrant expression of the S1P regulating enzymes, SPHK1 and SGPL1, contributes to a migratory phenotype in OSCC mediated through S1PR2. Sci. Rep..

[28] Ferreira LM, Hebrant A, Dumont JE (2012). Metabolic reprogramming of the tumor. Oncogene.

[29] Sijens PE, Levendag PC, Vecht CJ, van Dijk P, Oudkerk M (1996). 1H MR spectroscopy detection of lipids and lactate in metastatic brain tumors. Nmr. Biomed..

[30] Metser U (2006). 18F-FDG PET/CT in the evaluation of adrenal masses. J. Nucl. Med..

[31] Le TT, Huff TB, Cheng JX (2009). Coherent anti-Stokes Raman scattering imaging of lipids in cancer metastasis. BMC. Cancer.

[32] Mitra R, Chao O, Urasaki Y, Goodman OB, Le TT (2012). Detection of lipid-rich prostate circulating tumour cells with coherent anti-Stokes Raman scattering microscopy. BMC. Cancer.

[33] Mitra R, Goodman OB, Le TT (2014). Enhanced detection of metastatic prostate cancer cells in human plasma with lipid bodies staining. BMC. Cancer.

[34] Yang P (2012). Arachidonic acid metabolism in human prostate cancer. Int. J. Oncol..

[35] Wang D, Dubois RN (2010). Eicosanoids and cancer. Nat. Rev. Cancer.

[36] Cathcart MC, Lysaght J, Pidgeon GP (2011). Eicosanoid signalling pathways in the development and progression of colorectal cancer: novel approaches for prevention/intervention. Cancer Metastas. Rev..

[37] Zhou J (2005). Interactions between prostaglandin E(2), liver receptor homologue-1, and aromatase in breast cancer. Cancer Res..

[38] Wang D, Buchanan FG, Wang H, Dey SK, DuBois RN (2005). Prostaglandin E2 enhances intestinal adenoma growth via activation of the Ras-mitogen-activated protein kinase cascade. Cancer Res..

[39] Castellone MD, Teramoto H, Williams BO, Druey KM, Gutkind JS (2005). Prostaglandin E2 promotes colon cancer cell growth through a Gs-axin-beta-catenin signaling axis. Science.

[40] Pan MR, Hou MF, Chang HC, Hung WC (2008). Cyclooxygenase-2 up-regulates CCR7 via EP2/EP4 receptor signaling pathways to enhance lymphatic invasion of breast cancer cells. J. Biol. Chem..

[41] Buchanan FG (2006). Role of beta-arrestin 1 in the metastatic progression of colorectal cancer. Proc. Natl. Acad. Sci. USA.

[42] Iitaka D, Moodley S, Shimizu H, Bai XH, Liu M (2015). PKCdelta-iPLA2-PGE2-PPARgamma signaling cascade mediates TNF-alpha induced Claudin 1 expression in human lung carcinoma cells. Cell. Signal..

[43] Panigrahy D, Greene ER, Pozzi A, Wang DW, Zeldin DC (2011). EET signaling in cancer. Cancer Metastas. Rev..

[44] Wang D, Dubois RN (2012). Epoxyeicosatrienoic acids: a double-edged sword in cardiovascular diseases and cancer. J. Clin. Invest..

[45] Zhang SY, Surapureddi S, Coulter S, Ferguson SS, Goldstein JA (2012). Human CYP2C8 is post-transcriptionally regulated by microRNAs 103 and 107 in human liver. Mol. Pharmacol..

[46] Liu L (2011). Epoxyeicosatrienoic acids attenuate reactive oxygen species level, mitochondrial dysfunction, caspase activation, and apoptosis in carcinoma cells treated with arsenic trioxide. J. Pharmacol. Exp. Ther..

[47] Jiang JG (2007). Cytochrome p450 epoxygenase promotes human cancer metastasis. Cancer Res..

[48] Wei X (2014). Elevated 14,15- epoxyeicosatrienoic acid by increasing of cytochrome P450 2C8, 2C9, and 2J2 and decreasing of soluble epoxide hydrolase associated with aggressiveness of human breast cancer. BMC. Cancer.

[49] Panigrahy D (2012). Epoxyeicosanoids stimulate multiorgan metastasis and tumor dormancy escape in mice. J. Clin. Invest..

[50] Ooms LM (2015). The Inositol Polyphosphate 5-phosphatase PIPP regulates AKT1-dependent breast cancer growth and metastasis. Cancer Cell..

[51] Sengelaub CA, Navrazhina K, Ross JB, Halberg N, Tavazoie SFPTPRN2 (2016). and PLCbeta1 promote metastatic breast cancer cell migration through PI(4,5)P2-dependent actin remodeling. EMBO J..

[52] Vicinanza M, D’Angelo G, Di Campli A, De Matteis MA (2008). Function and dysfunction of the PI system in membrane trafficking. EMBO J..

[53] Balla T (2013). Phosphoinositides: tiny lipids with giant impact on cell regulation. Physiol. Rev..

[54] Fritz G, Kaina B (2006). Rho GTPases: promising cellular targets for novel anticancer drugs. Curr. Cancer Drug. Targets.

[55] Santiskulvong C (2011). Dual targeting of phosphoinositide 3-kinase and mammalian target of rapamycin using NVP-BEZ235 as a novel therapeutic approach in human ovarian carcinoma. Clin. Cancer Res..

[56] Manara MC (2010). NVP-BEZ235 as a new therapeutic option for sarcomas. Clin. Cancer Res..

[57] Milstien S, Spiegel S (2006). Targeting sphingosine-1-phosphate: a novel avenue for cancer therapeutics. Cancer Cell..

[58] Presa N (2016). Regulation of cell migration and inflammation by ceramide 1-phosphate. Biochim. Biophys. Acta.

[59] Morad SA (2016). Short-chain ceramides depress integrin cell surface expression and function in colorectal cancer cells. Cancer Lett..

[60] Le Stunff H, Galve-Roperh I, Peterson C, Milstien S, Spiegel S (2002). Sphingosine-1-phosphate phosphohydrolase in regulation of sphingolipid metabolism and apoptosis. J. Cell. Biol..

[61] Maceyka M, Harikumar KB, Milstien S, Spiegel S (2012). Sphingosine-1-phosphate signaling and its role in disease. Trends Cell Biol..

[62] Orr Gandy KA, Obeid LM (2013). Targeting the sphingosine kinase/sphingosine 1-phosphate pathway in disease: review of sphingosine kinase inhibitors. Biochim. Biophys. Acta.

[63] Pyne NJ, Pyne S (2010). Sphingosine 1-phosphate and cancer. Nat. Rev. Cancer.

[64] Lee H (2010). STAT3-induced S1PR1 expression is crucial for persistent STAT3 activation in tumors. Nat. Med..

[65] Furuhashi M, Hotamisligil GS (2008). Fatty acid-binding proteins: role in metabolic diseases and potential as drug targets. Nat. Rev. Drug. Discov..

[66] Ku CY, Liu YH, Lin HY, Lu SC, Lin JY (2016). Liver fatty acid-binding protein (L-FABP) promotes cellular angiogenesis and migration in hepatocellular carcinoma. Oncotarget.

[67] Tang Z (2016). Elevated expression of FABP3 and FABP4 cooperatively correlates with poor prognosis in non-small cell lung cancer (NSCLC). Oncotarget.

[68] Powell CA (2015). Fatty acid binding protein 5 promotes metastatic potential of triple negative breast cancer cells through enhancing epidermal growth factor receptor stability. Oncotarget.

[69] Rodic S, Mihalcioiu C, Saleh RR (2014). Detection methods of circulating tumor cells in cutaneous melanoma: a systematic review. Crit. Rev. Oncol. Hematol..

[70] Raghow R, Yellaturu C, Deng X, Park EA, Elam MB (2008). SREBPs: the crossroads of physiological and pathological lipid homeostasis. Trends Endocrinol. Metab..

[71] Qu Q, Zeng F, Liu X, Wang QJ, Deng F (2016). Fatty acid oxidation and carnitine palmitoyltransferase I: emerging therapeutic targets in cancer. Cell Death Dis..

[72] Li Y (2011). AMPK phosphorylates and inhibits SREBP activity to attenuate hepatic steatosis and atherosclerosis in diet-induced insulin-resistant mice. Cell. Metab..

[73] Warburg O (1956). On the origin of cancer cells. Science.

[74] Huang WC, Li X, Liu J, Lin J, Chung LW (2012). Activation of androgen receptor, lipogenesis, and oxidative stress converged by SREBP-1 is responsible for regulating growth and progression of prostate cancer cells. Mol. Cancer Res..

[75] Li C (2014). SREBP-1 has a prognostic role and contributes to invasion and metastasis in human hepatocellular carcinoma. Int. J. Mol. Sci..

[76] Bao J (2016). SREBP-1 is an independent prognostic marker and promotes invasion and migration in breast cancer. Oncol. Lett..

[77] Wang Y (2015). PD-L1 induces epithelial-to-mesenchymal transition via activating SREBP-1c in renal cell carcinoma. Med. Oncol..

[78] Li X (2016). SREBP-2 promotes stem cell-like properties and metastasis by transcriptional activation of c-Myc in prostate cancer. Oncotarget.

[79] Li X, Chen YT, Hu P, Huang WC (2014). Fatostatin displays high antitumor activity in prostate cancer by blocking SREBP-regulated metabolic pathways and androgen receptor signaling. Mol. Cancer Ther..

[80] Manning BD, Cantley LC (2007). AKT/PKB signaling: navigating downstream. Cell.

[81] Manning BD, Toker A (2017). AKT/PKB signaling: navigating the network. Cell.

[82] Li S, Brown MS, Goldstein JL (2010). Bifurcation of insulin signaling pathway in rat liver: mTORC1 required for stimulation of lipogenesis, but not inhibition of gluconeogenesis. Proc. Natl. Acad. Sci. USA.

[83] Wang Y, Viscarra J, Kim SJ, Sul HS (2015). Transcriptional regulation of hepatic lipogenesis. Nat. Rev. Mol. Cell Biol..

[84] Fu P (2016). Role of sphingosine kinase 1 and S1P transporter Spns2 in HGF-mediated lamellipodia formation in lung endothelium. J. Biol. Chem..

[85] Herzig Sébastien, Shaw Reuben J. (2017). AMPK: guardian of metabolism and mitochondrial homeostasis. Nature Reviews Molecular Cell Biology.

[86] Ahmadian M (2011). Desnutrin/ATGL is regulated by AMPK and is required for a brown adipose phenotype. Cell. Metab..

[87] Gwinn DM (2008). AMPK phosphorylation of raptor mediates a metabolic checkpoint. Mol. Cell.

[88] Wu N (2013). AMPK-dependent degradation of TXNIP upon energy stress leads to enhanced glucose uptake via GLUT1. Mol. Cell.

[89] Egan DF (2011). Phosphorylation of ULK1 (hATG1) by AMP-activated protein kinase connects energy sensing to mitophagy. Science.

[90] Toyama EQ (2016). Metabolism. AMP activated protein kinase mediated mitochondrial fission in response to energy stress.. Science.

[91] Day EA, Ford RJ, Steinberg GR (2017). AMPK as a therapeutic target for treating metabolic diseases. Trends Endocrinol. Metab..

[92] Goransson O (2007). Mechanism of action of A-769662, a valuable tool for activation of AMP-activated protein kinase. J. Biol. Chem..

[93] Rodgers JT, Lerin C, Gerhart-Hines Z, Puigserver P (2008). Metabolic adaptations through the PGC-1 alpha and SIRT1 pathways. FEBS Lett..

[94] Sundaresan NR (2011). The deacetylase SIRT1 promotes membrane localization and activation of Akt and PDK1 during tumorigenesis and cardiac hypertrophy. Sci. Signal..

[95] Gallardo-Montejano VI (2016). Nuclear Perilipin 5 integrates lipid droplet lipolysis with PGC-1alpha/SIRT1-dependent transcriptional regulation of mitochondrial function. Nat. Commun..

[96] Tan ZQ (2016). The Role of PGC1 alpha in cancer metabolism and its therapeutic implications. Mol. Cancer Ther..

[97] Villena JA (2015). New insights into PGC-1 coactivators: redefining their role in the regulation of mitochondrial function and beyond. FEBS. J..

[98] Hao C (2014). Overexpression of SIRT1 promotes metastasis through epithelial-mesenchymal transition in hepatocellular carcinoma. BMC. Cancer.

[99] LeBleu VS (2014). PGC-1alpha mediates mitochondrial biogenesis and oxidative phosphorylation in cancer cells to promote metastasis. Nat. Cell Biol..

[100] Li Y (2016). SIRT1 facilitates hepatocellular carcinoma metastasis by promoting PGC-1alpha-mediated mitochondrial biogenesis. Oncotarget.

[101] Nagrath S (2007). Isolation of rare circulating tumour cells in cancer patients by microchip technology. Nature.

[102] Andreopoulou E (2012). Comparison of assay methods for detection of circulating tumor cells in metastatic breast cancer: AdnaGen AdnaTest BreastCancer Select/Detect versus Veridex CellSearch system. Int. J. Cancer.

[103] Tan SJ, Yobas L, Lee GY, Ong CN, Lim CT (2009). Microdevice for the isolation and enumeration of cancer cells from blood. Biomed. Micro..

[104] Bobrie A, Colombo M, Raposo G, Thery C (2011). Exosome secretion: molecular mechanisms and roles in immune responses. Traffic.

[105] Colombo M, Raposo G, Thery C (2014). Biogenesis, secretion, and intercellular interactions of exosomes and other extracellular vesicles. Annu. Rev. Cell. Dev. Biol..

[106] Kaiser J (2016). Malignant messengers. Science.

[107] Skotland T, Sandvig K, Llorente A (2017). Lipids in exosomes: Current knowledge and the way forward. Prog. Lipid Res..

[108] Ghossoub R (2014). Syntenin-ALIX exosome biogenesis and budding into multivesicular bodies are controlled by ARF6 and PLD2. Nat. Commun..

[109] Trajkovic K (2008). Ceramide triggers budding of exosome vesicles into multivesicular endosomes. Science.

[110] Mittelbrunn M (2011). Unidirectional transfer of microRNA-loaded exosomes from T cells to antigen-presenting cells. Nat. Commun..

[111] Hessvik NP (2016). PIKfyve inhibition increases exosome release and induces secretory autophagy. Cell. Mol. Life Sci..

[112] Rak J (2013). Extracellular vesicles—biomarkers and effectors of the cellular interactome in cancer. Front. Pharmacol..

[113] Ferguson SW, Nguyen J (2016). Exosomes as therapeutics: the implications of molecular composition and exosomal heterogeneity. J. Control Release.

[114] Fais S (2016). Evidence-based clinical use of nanoscale extracellular vesicles in nanomedicine. ACS Nano.

[115] Skotland T (2017). Molecular lipid species in urinary exosomes as potential prostate cancer biomarkers. Eur. J. Cancer.

[116] Beloribi-Djefaflia S, Siret C, Lombardo D (2015). Exosomal lipids induce human pancreatic tumoral MiaPaCa-2 cells resistance through the CXCR4-SDF-1alpha signaling axis. Oncoscience.

